# Atraumatic Myositis Ossificans of the Digastric Muscle: Case Report

**DOI:** 10.1002/ccr3.71312

**Published:** 2025-10-15

**Authors:** Samuel Tschopp, Urs Borner, Florian Dammann, Roland Giger

**Affiliations:** ^1^ Department of Otorhinolaryngology, Head and Neck Surgery Inselspital, Bern University Hospital and University of Bern Bern Switzerland; ^2^ Department of Diagnostic, Interventional and Pediatric Radiology Inselspital, Bern University Hospital and University of Bern Bern Switzerland

**Keywords:** bruxism, heterotopic ossification, myositis ossificans, myositis ossificans circumscripta, submental mass

## Abstract

Atraumatic myositis ossificans in the head and neck is rare and diagnostically challenging. Its rapid growth, frequently inconclusive biopsies, and nonspecific imaging findings can mimic malignancy, such as sarcoma, leading to potential misdiagnosis. Awareness of this entity is crucial for clinicians to avoid unnecessary overtreatment and ensure appropriate management.

## 1 Introduction

Myositis ossificans is a benign condition characterized by heterotopic ossification, predominantly occurring in the extremities following trauma. In this case report, we present the first described case of atraumatic myositis ossificans in the digastric muscle. This form of heterotopic ossification can pose significant diagnostic challenges due to its atypical location and potential resemblance to malignant processes [1]. Written informed consent was obtained from the patient for publication of this case report and any accompanying images or data.

## 2 Case History

**FIGURE 1:**
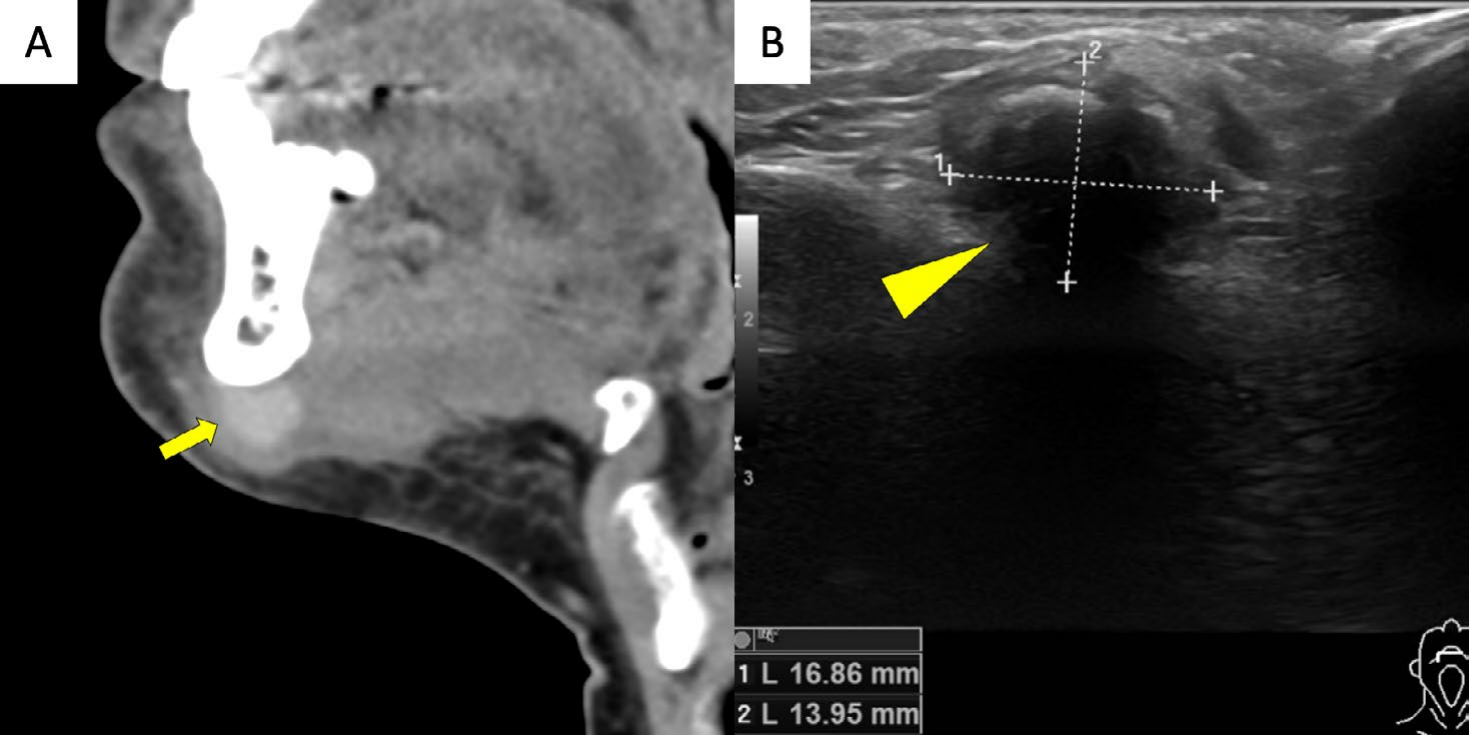
Computed tomography imaging and ultrasonographic imaging. (A) Sagittal view shows a contrast‐enhancing mass (open arrow) abutting the mandible with suspected infiltration of the digastric muscle and thickening of the adjacent platysma, but without evidence of sclerotic changes. (B) In the ultrasound, a submental mass (arrowhead) with an irregular hyperechogenic border is seen within the digastric muscle, accompanied by a posterior acoustic shadow.

A 53‐year‐old man presented with a firm, round, submental midline mass measuring approximately 2 cm. He first noticed a hard growth below the mandible and experienced pain during mouth opening. His medical history included well‐controlled arterial hypertension and gout. Additionally, he reported intense bruxism, managed with an oral device. He had no history of trauma or recent dental procedures. Two weeks after noticing the mass, he consulted an otorhinolaryngologist, who performed a fine‐needle aspiration and ordered a computed tomography scan. Cytology revealed atypical spindle cell proliferation. The computed tomography scan (Figure 1A) showed an 18 mm contrast‐enhancing mass abutting the mandible, with suspected infiltration of the digastric muscle, without additional pathologies.

Four weeks after symptom onset, the patient was referred to our institution, reporting slow growth of the mass. Ultrasound (Figure 1B) revealed an irregularly shaped tumor with a hyperechogenic border within the digastric muscle, with a posterior acoustic shadow. A core‐needle biopsy was performed, demonstrating collagen fiber‐rich spindle cell proliferation, skeletal muscle, and vascularized fatty connective tissue. No malignancy was detected, but due to the lesion's hard consistency, sampling of the center was not possible with a core‐needle biopsy.

## 3 Differential Diagnosis, Investigations, and Treatment

**FIGURE 2:**
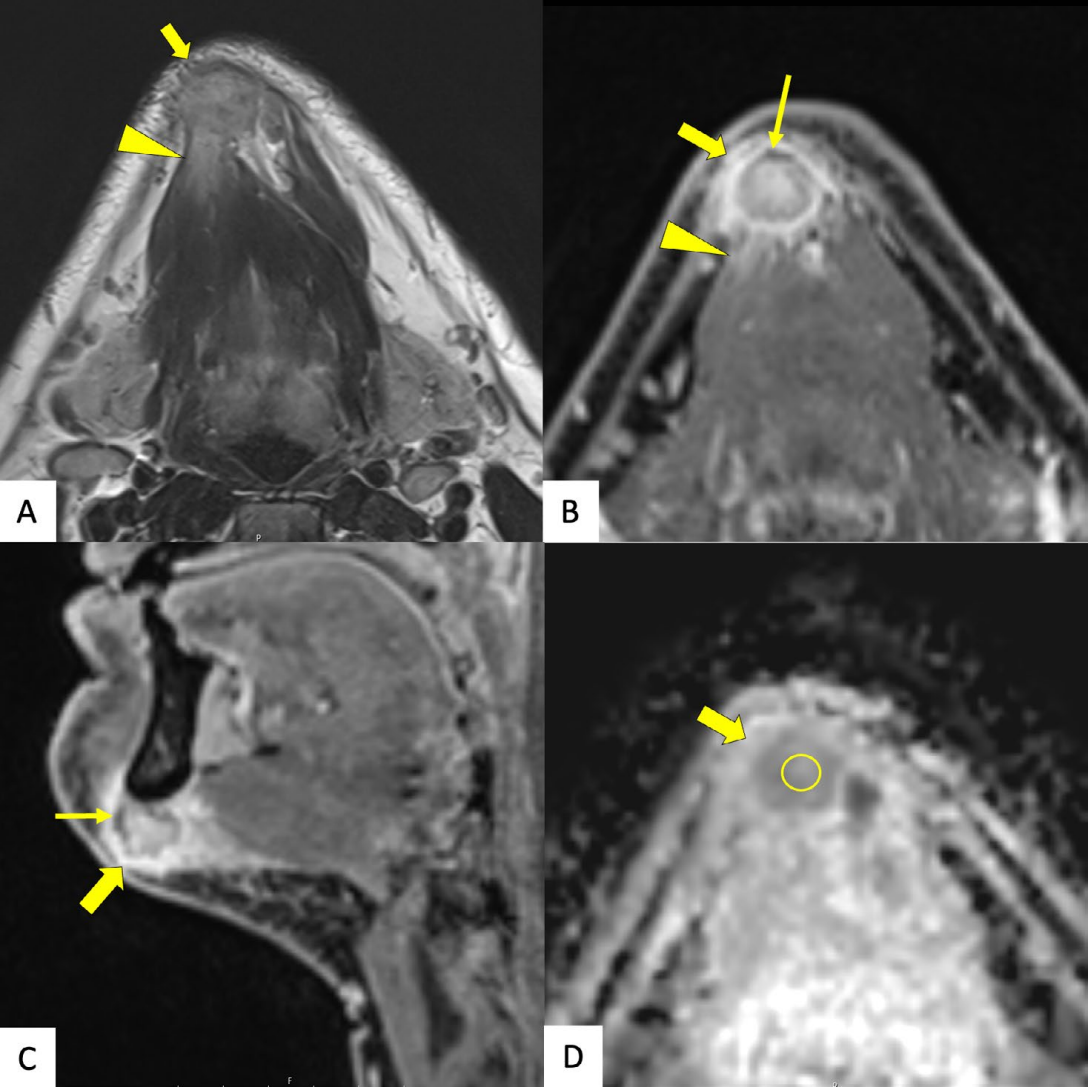
Magnetic resonance imaging. (A) Transverse T2‐weighted images, (B) contrast enhanced high resolution T1‐weighted images in transversal and (C) in sagittal planes show a well‐defined tumor (open arrow) with unsharp infiltration of the anterior belly of the right digastric muscle (arrowhead). T1‐weighted images, which firmly correspond to the gross specimen images (Figure 3), additionally show linear areas of low signal intensity corresponding to calcifications (arrow), which were not detected by CT. (D) In diffusion weighted imaging, lowest calculated ADC values of 1049 mm^2^/s were identified in the center of the lesion (circle), indicating a benign nature of the lesion.

To further characterize the mass, magnetic resonance imaging (Figure 2) was performed approximately 6 weeks after symptom onset. The imaging confirmed a stable‐sized mass in the right submental region with features highly suspicious for malignancy. While the lesion appeared to infiltrate the adjacent musculature and periosteum of the mandible, no bony invasion was evident. After a multidisciplinary discussion with clinical and radiological suspicion of malignancy, adequate surgical excision was recommended, and the patient consented.

**FIGURE 3:**
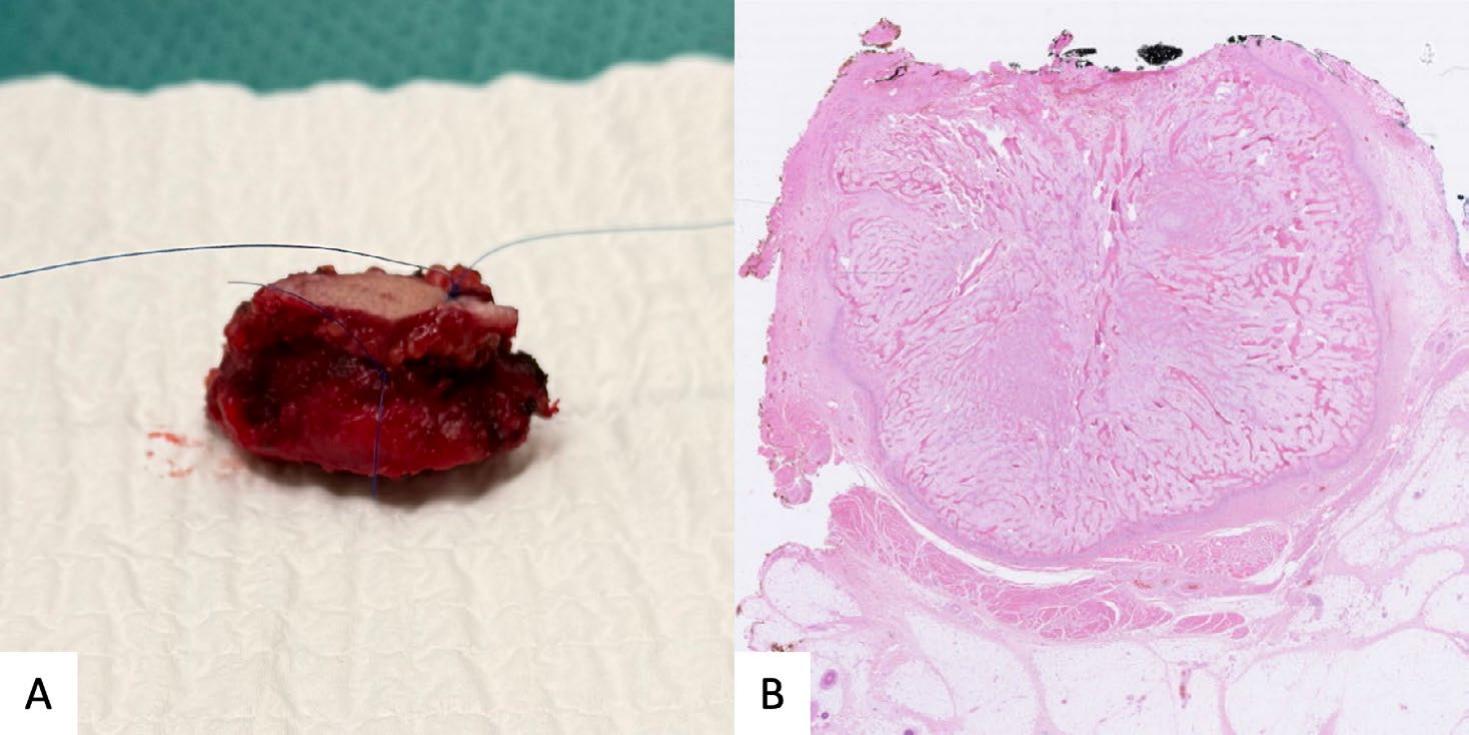
Excised myositis ossificans. (A) Superior view of the excised mass with a small margin of healthy soft tissue and muscle. (B) Histopathologic slide of the myositis ossificans stained with hematoxylin and eosin demonstrating a characteristic zonal pattern. The peripheral zone consists of mature lamellar bone, the intermediate zone contains fibroblasts, osteoblasts, and osteoid, while the inner core is comprised of a myofibroblastic inner core with mild pleomorphism.

Under general anesthesia, a horizontal submental incision was made, and the mass was carefully dissected. The lesion was rigid and incompressible and adhered to the inferior border of the mandible. It was freed from the mandible with resection of the periosteum and excised with a macroscopic margin of at least 1 cm healthy tissue. No bony invasion was observed. The excised specimen (Figure 3A) was assessed for completeness and sent for histopathological analysis. The patient recovered uneventfully, with no functional deficits.

## 4 Outcome and Follow‐Up

Histopathology examination (Figure 3B) confirmed the diagnosis of myositis ossificans. The peripheral zone consisted of lamellar bone without atypia, while the inner core displayed myofibroblastic proliferation with mild pleomorphism and mitotic activity. An intermediate zone contained a mixture of fibroblasts and osteoblasts along with irregular osteoid deposition.

## 5 Discussion

Myositis ossificans circumscripta, also known as traumatic myositis ossificans, is a form of heterotopic ossification that most commonly occurs in the extremities following trauma or orthopedic surgery [1, 2]. Approximately 30% of hip surgeries are estimated to result in heterotopic ossification [3, 4]. In rare cases, myositis ossificans develops without identifiable inciting injury [1, 5]. In the head and neck, it has been described primarily in the masseter and pterygoid muscles, often following dental procedures [6, 7]. Progressive myositis ossificans is an inherited condition affecting multiple sites and is not the focus of this case report [1].

The pathophysiology of myositis ossificans remains incompletely understood [2, 4, 8]. Typically, the process begins within weeks after an injury, likely due to local cellular dysregulation. Following an initial inflammatory response, stem cells differentiate into chondrocytes and osteoblasts, initiating ectopic bone formation [2, 4, 8]. Ossification progresses in distinct phases, producing a characteristic zonal histology: a peripheral shell of mature bone forms first, followed by progressive calcification toward the center through endochondral ossification. Over time, the lesion consolidates into mature bone [1].

In the extremities, heterotopic ossifications often present with a restricted range of motion and pain during movement due to mechanical interference [1]. However, diagnosis can be challenging, as myositis ossificans can closely mimic malignancies such as sarcomas both clinically, radiologically, and even histologically in some cases [9, 10]. Magnetic resonance imaging is the preferred imaging modality, though ultrasonography is commonly used as an initial assessment [9]. Although PET/CT can demonstrate metabolic activity and differentiate active from mature ossification, its frequent intense FDG uptake mimicking malignancy limits its diagnostic utility in myositis ossificans [11, 12]. Given the nonspecific imaging findings, a biopsy is often necessary for diagnosis. Fine‐needle aspiration, core‐needle biopsy, or excisional biopsy have all been utilized in diagnosing myositis ossificans [1].

The standard management approach depends on symptom severity. Asymptomatic patients may be observed, while mildly symptomatic patients benefit from supportive care and physical therapy. Surgical excision remains the treatment for symptomatic or diagnostically uncertain cases [6, 7]. In our patient, excision was recommended to confirm the diagnosis and alleviate symptoms.

This case of myositis ossificans in the digastric muscle presents several unique aspects.

First, the unusual location. Heterotopic ossification predominantly affects the extremities, particularly the upper arms and thighs. In the head and neck, previously reported cases have involved the masseter and pterygoid muscles, typically following trauma or dental procedures [6, 7]. To our knowledge, this is the first documented case of submental myositis ossificans in the digastric muscle.

Second, the absence of known trauma. Myositis ossificans is usually associated with direct trauma or surgical intervention [1, 2, 3, 4, 6, 7]. However, our patient had no history of trauma. Instead, he reported severe bruxism, which may have contributed to chronic microtrauma and subsequent inflammatory changes, potentially triggering heterotopic ossification.

Third, the diagnostic uncertainty. The rapid growth of the lesion, inconclusive fine‐needle and core‐needle biopsies, and nonspecific imaging findings initially raised concerns for malignancy, especially sarcoma. The fine‐needle aspiration cytology revealed atypical spindle cell proliferation, while the core‐needle biopsy was limited by the hard consistency of the lesion, making adequate sampling challenging. The computed tomography showed no typical ossifications, possibly because it was performed early before bone formation. The increased apparent diffusion coefficient in magnetic resonance imaging suggests benign pathology but lacks specificity, underscoring the difficult distinction from malignant processes.

## 6 Conclusion

Atraumatic myositis ossificans in the head and neck is rare and diagnostically challenging. Its rapid growth, frequently inconclusive biopsies, and nonspecific imaging findings can mimic malignancy, such as sarcoma, leading to potential misdiagnosis. Awareness of this entity is crucial for clinicians to avoid unnecessary overtreatment and ensure appropriate management.

## Author Contributions

**Samuel Tschopp:** conceptualization, data curation, methodology, project administration, visualization, writing – original draft, writing – review and editing. **Urs Borner:** conceptualization, data curation, methodology, supervision, visualization, writing – review and editing. **Florian Dammann:** conceptualization, data curation, methodology, resources, supervision, visualization, writing – review and editing. **Roland Giger:** conceptualization, data curation, resources, supervision, visualization, writing – review and editing.

## Consent

Written informed consent was obtained from the patient for publication of this case report and any accompanying images or data. All efforts were made to ensure patient anonymity.

## Conflicts of Interest

The authors declare no conflicts of interest.

## Data Availability

The data from this case report are available from the corresponding author upon reasonable request.
